# Predição de Re-hospitalização em Cirurgias de Revascularização Cardíaca

**DOI:** 10.36660/abc.20240706

**Published:** 2025-03-06

**Authors:** Antônio Rafael Alves de Sá, Victoria Alves Prado, Mateus Augusto Felix de Melo, Pâmela Marillac Rodrigues Feijó de Melo, Maria Clara Filgueira Borba, Achilles de Souza Andrade, Jorge Fernando Pereira Silva, Johnnatas Mikael Lopes

**Affiliations:** 1 Universidade Federal do Vale do São Francisco Paulo Afonso BA Brasil Universidade Federal do Vale do São Francisco (UNIVASF), Paulo Afonso, BA – Brasil; 2 Centro Universitário Maurício de Nassau Recife PE Brasil Centro Universitário Maurício de Nassau (Uninassau), Recife, PE – Brasil; 3 Universidade Federal da Paraíba João Pessoa PB Brasil Universidade Federal da Paraíba, João Pessoa, PB – Brasil

**Keywords:** Revascularização Miocárdica, Readmissão do Paciente, Estudos de Coortes

A doença arterial coronariana, uma doença cardiovascular onde há obstrução do fluxo sanguíneo dos vasos do miocárdio, causa grande número de óbitos no Brasil, anualmente. A cirurgia de revascularização miocárdica, associada a medicamentos antiplaquetários, hipolipemiantes e bloqueadores do SRAA, mostra-se como a melhor opção para tratar e prevenir novos casos de obstrução do fluxo vascular, permitindo, assim, restaurar a função ventricular, reduzir a angina e prevenir casos de infarto agudo do miocárdio. Assim, tem importância clínica e cirúrgica, pois permite tratar obstruções múltiplas e melhorar a sobrevida dos pacientes.^1 , 2^

A intervenção coronária percutânea (ICP) é o procedimento de revascularização mais frequentemente utilizado em pacientes com doença coronariana, sendo realizada tanto de forma eletiva quanto em situações agudas.^3^ Re-hospitalizações não planejadas podem ser consideradas um resultado adverso para os pacientes e podem estar relacionadas a complicações do procedimento de ICP ou do tratamento hospitalar. Por isso, elas são frequentemente utilizadas como um indicador da qualidade do atendimento prestado. Estima-se que 1 em cada 7 pacientes submetidos a ICP são readmitidos em 30 dias.^3^

Analisando os dados da publicação “Preditores de Readmissão Hospitalar até 30 Dias de CRM em Banco de Dados Multicêntrico: Estudo de Coorte Transversal”, identificamos um potencial de informações de extrema relevância para a prática clínica e a gestão de unidades de cirurgia cardíaca.^4^ Contudo, há elementos metodológicos que necessitam ser destacados a fim de minimizar vieses em estudos de síntese como as revisões com metanálise.

A primeira delas é que o desenho se trata de estudo de coorte retrospectivo ou histórico. Não existe esta classificação de estudo de coorte transversal. O segundo aspecto metodológico relevante, e talvez o principal, é a utilização da técnica de regressão logística (RL) para estudos de coorte.^5^ Diferentemente dos desenhos de caso-controle, o OR estimado pela RL em estudos de coortes tanto prospectiva como retrospectiva superdimensiona os intervalos de confiança, o que pode tornar variáveis expositoras não significativas quando suas estimativas pontuais estão próximas de 1,^5^ como é o caso da variável arritmia cardíaca (OR=1,04; IC:0,997-1,085).^5^ Apesar dos autores não explicitarem no método todas as variáveis independentes analisadas para o modelo, é recomendado apresentar todo o processo de construção do modelo com as variáveis significativas ou não.

O terceiro elemento metodológico que merece destaque é o efeito *cluster* que os diferentes centros clínicos fornecedores de dados podem gerar na ocorrência dos desfechos. Isto ocorre porque pode existir aspectos organizacionais nas instituições que favorecem ou não a readmissão hospitalar em pós-cirúrgico de revascularização. Todos os estudos multicêntricos precisam avaliar o efeito *cluster* .^6^ Para solucionar o segundo e terceiro destaques metodológicos, os dados precisam ser analisados por meio de técnicas de Modelos Lineares Generalizáveis como GEE ou Modelos Mistos, aplicando distribuição de Poisson.^6^ Assim, é possível controlar o efeito do superdimensionamento dos IC e o efeito *cluster* .

## References

[1] Lucca MB, Fuchs FC, Almeida AS, Wainstein MV, Fuchs FD, Fuchs SC (2023). Secondary Pharmacological Prevention of Coronary Artery Disease among Patients Submitted to Clinical Management, Percutaneous Coronary Intervention, or Coronary Artery Bypass Graft Surgery. Arq Bras Cardiol.

[2] Lacava L, Freitas FL, Borgomoni GB, Silva PGMBE, Nakazone MA, Campagnucci VP (2024). More Hospital Complications in Women after Cabg Even for Reduced Surgical Times: Call to Action for Equity in Quality Improvement. Arq Bras Cardiol.

[3] Kwok CS, Narain A, Pacha HM, Lo TS, Holroyd EW, Alraies MC (2020). Readmissions to Hospital after Percutaneous Coronary Intervention: A Systematic Review and Meta-Analysis of Factors Associated with Readmissions. Cardiovasc Revasc Med.

[4] Silva RAGE, Borgomoni GB, Freitas FL, Maia ADS, Vale CF, Pereira EDS (2024). Predictors of 30-Day Hospital Readmission Following CABG in a Multicenter Database: A Cross-Sectional Study. Arq Bras Cardiol.

[5] Knol MJ, Le Cessie S, Algra A, Vandenbroucke JP, Groenwold RH (2012). Overestimation of Risk Ratios by Odds Ratios in Trials and Cohort Studies: Alternatives to Logistic Regression. CMAJ.

[6] Conroy EJ, Blazeby JM, Burnside G, Cook JA, Gamble C (2020). Managing Clustering Effects and Learning Effects in the Design and Analysis of Multicentre Randomised Trials: A Survey to Establish Current Practice. Trials.

