# Supplementary material for: Predição de Re-hospitalização em Cirurgias de Revascularização Cardíaca
Source: Arq Bras Cardiol. 2025 Mar 6;122(2):e20240706. [Article in Portuguese] doi: 10.36660/abc.20240706 (PMC12080594; doi:10.36660/abc.20240706)
Supplement: Translation

# Translation: Prediction of Rehospitalization in Cardiac Bypass Surgery

**DOI:** 10.36660/abc.20240706i

**Authors:** Antônio Rafael Alves de Sá, Victoria Alves Prado, Mateus Augusto Felix de Melo, Pâmela Marillac Rodrigues Feijó de Melo, Maria Clara Filgueira Borba, Achilles de Souza Andrade, Jorge Fernando Pereira Silva, Johnnatas Mikael Lopes

**Affiliations:** 1 Brazil Universidade Federal do Vale do São Francisco (UNIVASF), Paulo Afonso, BA – Brazil; 2 Brazil Centro Universitário Maurício de Nassau (Uninassau), Recife, PE – Brazil; 3 Brazil Universidade Federal da Paraíba, João Pessoa, PB – Brazil

**Keywords:** Myocardial Revascularization, Patient Readmission, Cohort Studies

Coronary artery disease, a cardiovascular disease in which there is obstruction of blood flow in the myocardial vessels, causes a large number of deaths in Brazil every year. Myocardial revascularization surgery, associated with antiplatelet, lipid-lowering, and RAAS blocker medications, is the best option for treating and preventing new cases of vascular flow obstruction, thus allowing the restoration of ventricular function, reduction of angina and prevention of acute myocardial infarction. Thus, it has clinical and surgical importance, as it allows the treatment of multiple obstructions and improves patient survival.^1 , 2^

Percutaneous coronary intervention (PCI) is the most frequently used revascularization procedure in patients with coronary disease and is performed both electively and in acute situations.^3^Unplanned rehospitalizations can be considered an adverse outcome for patients and may be related to complications of the PCI procedure or hospital treatment. Therefore, they are often used as an indicator of the quality of care provided. It is estimated that 1 in 7 patients undergoing PCI are readmitted within 30 days.^3^

Analyzing the data from the publication “Predictors of 30-Day Hospital Readmission Following CABG in a Multicenter Database: A Cross-Sectional Study”,^4^we

identified potential information of extreme relevance for clinical practice and the management of cardiac surgery units. However, there are methodological elements that need to be highlighted in order to minimize biases in synthesis studies, such as reviews with meta-analysis.

The first of these is that the design is a retrospective or historical cohort study. There is no such classification for a cross-sectional cohort study. The second relevant methodological aspect, and perhaps the main one, is the use of the logistic regression (LR) technique for cohort studies.^5^Unlike case-control designs, the OR estimated by LR in both prospective and retrospective cohort studies oversizes the confidence intervals, which can make exposed variables non-significant when their point estimates are close to 1,^5^as is the case of the variable cardiac arrhythmia (OR=1.04; CI: 0.997-1.085).^5^Although the authors do not explicitly state in the method all the independent variables analyzed for the model, it is recommended to present the entire model construction process with the variables significant or not.

The third methodological element that deserves to be highlighted is the cluster effect that the different clinical centers providing data can generate in the occurrence of the outcomes. This occurs because there may be organizational aspects in the institutions that favor or not hospital readmission after revascularization surgery. All multicenter studies need to evaluate the cluster effect.^6^To solve the second and third methodological issues, the data need to be analyzed using Generalizable Linear Model techniques such as GEE or Mixed Models, applying Poisson distribution.^6^Thus, it is possible to control the effect of oversizing of CIs and the cluster effect.
